# A Dietary Treatment Improves Cerebral Blood Flow and Brain Connectivity in Aging apoE4 Mice

**DOI:** 10.1155/2016/6846721

**Published:** 2016-03-10

**Authors:** Maximilian Wiesmann, Valerio Zerbi, Diane Jansen, Roy Haast, Dieter Lütjohann, Laus M. Broersen, Arend Heerschap, Amanda J. Kiliaan

**Affiliations:** ^1^Department of Anatomy, Donders Institute for Brain, Cognition & Behaviour, Radboud University Medical Center, 6525 EZ Nijmegen, Netherlands; ^2^Department of Geriatric Medicine, Radboud University Medical Center, 6525 EZ Nijmegen, Netherlands; ^3^Department of Radiology and Nuclear Medicine, Radboud University Medical Center, 6525 EZ Nijmegen, Netherlands; ^4^Institute for Clinical Chemistry and Clinical Pharmacology, University of Bonn, Bonn, Germany; ^5^Nutricia Research, Advanced Medical Nutrition, Utrecht, Netherlands; ^6^UIPS, Utrecht University, Utrecht, Netherlands

## Abstract

APOE *ε*4 (apoE4) polymorphism is the main genetic determinant of sporadic Alzheimer's disease (AD). A dietary approach (Fortasyn) including docosahexaenoic acid, eicosapentaenoic acid, uridine, choline, phospholipids, folic acid, vitamins B12, B6, C, and E, and selenium has been proposed for dietary management of AD. We hypothesize that the diet could inhibit AD-like pathologies in apoE4 mice, specifically cerebrovascular and connectivity impairment. Moreover, we evaluated the diet effect on cerebral blood flow (CBF), functional connectivity (FC), gray/white matter integrity, and postsynaptic density in aging apoE4 mice. At 10–12 months, apoE4 mice did not display prominent pathological differences compared to wild-type (WT) mice. However, 16–18-month-old apoE4 mice revealed reduced CBF and accelerated synaptic loss. The diet increased cortical CBF and amount of synapses and improved white matter integrity and FC in both aging apoE4 and WT mice. We demonstrated that protective mechanisms on vascular and synapse health are enhanced by Fortasyn, independent of apoE genotype. We further showed the efficacy of a multimodal translational approach, including advanced MR neuroimaging, to study dietary intervention on brain structure and function in aging.

## 1. Introduction

Extensive research has been pursued in search of an effective therapy for Alzheimer's disease (AD). However, no treatment is yet available nor does it seem near. Preventive approaches have therefore consistently emerged as key policy priorities in recently formulated dementia strategies. These approaches include modification of health-compromising behavior such as lifestyle and dietary intake that may lead to AD. For example, the Mediterranean diet (high consumption of fruit, vegetables, and legumes, moderate consumption of fish, nuts, and olive oil as the main source of fats) has been associated with a reduced risk of AD [1, 2] and with a lower mortality [3, 4]. The mechanisms via which diet influences the onset and progression of AD pathology are still under investigation. One possible mode of action is the beneficial effect of nutrients, such as omega-3 long-chain polyunsaturated fatty acids (n3-LCPUFAs) on the vascular system [4, 5].

Herewith n3-LCPUFAs will target the very early, asymptomatic phase of the disease, in which (cerebro)vascular impairment is the strongest contributor to the onset and progression of neurodegenerative traits of typical AD and dementia in general [6]. n3-LCPUFAs may diminish severity of vascular risk factors, like atherosclerosis [7], high blood pressure [8], and other cardiovascular diseases [7, 9–11], which are risk factors for AD as well. Other nutrients may instead directly protect synaptic integrity. For instance, the formation of phosphatidylcholine, the most common phosphatide in the brain and a major component of the synaptic membrane, is enhanced due to presence of its precursors in the diet [12, 13]. Several preclinical studies confirmed these findings, showing that animals supplemented with the combination of these membrane precursors showed increased levels of brain phospholipids, dendritic spines, and neurite outgrowth, with beneficial effects on cognition [14–18]. Based on these findings, a novel multinutrient supplementation diet called Fortasyn, comprising n3-LCPUFAs docosahexaenoic acid (DHA) and eicosapentaenoic acid (EPA), besides other precursors and cofactors for membrane synthesis, such as uridine, choline, phospholipids, folic acid, vitamins B12, B6, C, and E, and selenium, has been proposed for the dietary management of AD [19]. To date, two randomized controlled clinical trials have shown improvements in the delayed verbal recall task and better cognitive performance in mild AD patients supplemented with this nutrient combination [20–22]. Although it has been recognized that Fortasyn addresses specific nutritional needs in early AD and that it improves functional connectivity as assessed by EEG [23], other processes by which Fortasyn may influence the pathophysiology of AD need to be further elucidated, and more studies are required to confirm its efficacy.

Some studies suggested an interaction of the cholesterol transporter apolipoprotein (apoE) with lipid-based dietary intervention [24–26]. ApoE is a 34 kDa glycoprotein that exists in three isoforms: apoE-*ε*2, apoE-*ε*3, and apoE-*ε*4, which differ by one or two amino acid residues 112 and 158 [27]. This small difference strongly affects the conformation and the structure of apoE and influences its ability to bind lipids, receptors, and amyloid-*β* (A*β*) [28]. ApoE polymorphic alleles have been identified as the main genetic determinants of AD risk; specifically, apoE-*ε*4 has been associated with increased toxicity and loss of neuroprotective function in the pathogenesis of Alzheimer's disease, dependent on or independent of A*β* accumulation [29]. Importantly, some of these processes are directly or indirectly linked to an impaired vascular system [30–32]. The development of an appropriate animal model, targeting the murine APOE gene for replacement with the human APOE-*ε*4 (*apoE-ε4/apoE4* mouse) [33], opened a window for new possibilities of characterizing the apoE-*ε*4 phenotype and of studying the effects of very early AD-like pathology development in relation with lipid-based treatment. ApoE4 carrier mice exhibit an altered lipid profile, with increased risk of atherosclerosis plaques formation [33]. Altered behavior and cognitive deficits have also been reported [34]. A previous study from our group showed that a long-term dietary intervention with the Fortasyn diet was able to reduce anxiety behavior in 10-month-old apoE4 mice [35]. Transgenic AD mice on the same diet showed restored cortical cerebral blood flow (CBF) and brain structural integrity compared to wild-type mice [17].

Following these promising findings, we hypothesize that a nutritional intervention with Fortasyn may be able to rescue or prevent the occurrence of early AD-like pathologies in apoE4 mice, such as cerebrovascular impairment and concomitant brain connectivity loss. To test this hypothesis, we evaluated the effect of the Fortasyn diet on cerebral and plasma levels of fatty acids and sterols, cerebral blood flow (CBF), gray and white matter integrity, functional connectivity (FC), and postsynaptic density during aging in 12- and 18-month-old apoE4 mice.

## 2. Materials and Methods

### 2.1. Animals

The apoE-*ε*4 founder mice were originally obtained from Taconic Transgenic Models (Hudson, NY, USA) and a colony was established at the Radboud University Medical Center (Radboudumc). ApoE4 mice were created by targeting the murine apoE gene for replacement with the human apoE-*ε*4 alleles cultured in E14TG2a embryonic stem (ES) cells as described previously [36]. Resulting chimeras were backcrossed to C57BL/6J mice for 8 generations. The line was derived by embryo transfer and is maintained by inbreeding homozygous mice. For the present study, male and female apoE4 breeder mice were used to generate homozygous apoE4 offspring (3rd generation).

C57BL/6J wild-type (WT) mice, obtained from our colony at the Radboudumc, were used as controls. Throughout the experiment animals were housed in groups of 2–7 mice per cage in a controlled environment, homogenously illuminated by normal fluorescent room light at 60 lux, with room temperature at 21°C, and an artificial 12 : 12 h light : dark cycle (lights on at 7 am). Food and water were available* ad libitum*.

The experiments were performed according to Dutch federal regulations for animal protection. The Veterinary Authority of the Radboudumc, Netherlands, approved all the protocols within this study (RU-DEC 2011-058).

### 2.2. Diets and Timeline of Experimental Design

**Figure 1:**
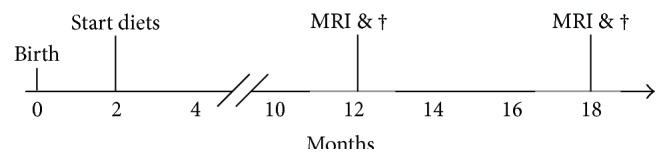
Timeline of experimental design. Starting from 2 months of age, mice were randomly divided into two groups; animals were fed either with control diet or with Fortasyn diet. The first group of mice underwent MR imaging (MRI) at 11 to 12 months of age and were sacrificed (†) immediately thereafter. The second group was sacrificed after MRI at 18 months of age.

Starting at 2 months of age, mice were randomly divided into two groups; animals were fed either with control diet or with Fortasyn diet that differed with respect to the presence of a specific combination of dietary precursors and cofactors, that is, uridine, docosahexaenoic acid, eicosapentaenoic acid, choline, phospholipids, folic acid, vitamins B12, B6, C, and E, and selenium (Table 1). Both diets were isocaloric and based on AIN-93 M [37] with 5% fat. The control diet contained 1.9% soy oil, 0.9% coconut oil, and 2.2% corn oil; the Fortasyn diet contained 0.1% coconut oil, 1.9% corn oil, and 3.0% fish oil. The Fortasyn-based diet contains a specific multinutrient composition comprising nucleotides, omega-3 PUFAs, choline, B vitamins, phospholipids, and antioxidants (Table 1). Diets were manufactured and pelleted by Ssniff (Soest, Germany) and stored at −20°C until use. The first group of mice underwent MR imaging at 11 (average age: 10.6 ± 0.1 months) to 12 (average age: 12.3 ± 0.1 months) months of age and was sacrificed immediately thereafter. The second group was scanned at 16 months of age (average age: 16.2 ± 0.1 months) and sacrificed at 18 months of age (average age: 17.9 ± 0.1 months). The timeline of the experimental design is illustrated in Figure 1. The sample size of minimal 6 mice (12-month: WT-control *n* = 9, WT-Fortasyn *n* = 9, apoE4-control *n* = 8, and apoE4-Fortasyn *n* = 10; 18-month: WT-control *n* = 10, WT-Fortasyn *n* = 10, apoE4-control *n* = 10, and apoE4-Fortasyn *n* = 6) per group was chosen based on formal calculation of power as described in the approved protocols (RU-DEC 2011-058).

### 2.3. MR Imaging

MRI measurements were performed on an 11.7 T BioSpec Avance III small animal MR system (Bruker BioSpin, Ettlingen, Germany) equipped with an actively shielded gradient set of 600 mT/m and operated by ParaVision 5.1 software between 8 am and 8 pm. We used a circular polarized volume resonator for signal transmission and an actively decoupled mouse brain quadrature surface coil for signal reception (Bruker BioSpin). During the MR experiments, low-dose isoflurane was used (3.5% for induction and ~1.5% for maintenance), slightly adjusted throughout the experiment to maintain a fast and stable breathing frequency (>130 bpm). The mice were placed in a stereotactic device in order to immobilize the head. Body temperature was measured with a rectal thermometer and maintained at 37°C by a heated air flow device.

After standard adjustments and shimming, gradient echo (GE) T_2_^*∗*^-weighted images covering the entire mouse brain were acquired for anatomical reference.

To study brain perfusion under resting conditions, we used a flow-sensitive alternating inversion recovery arterial spin labelling (FAIR ASL) technique [17, 38]. Briefly, fifteen images with increasing inversion times (TIs) (40 ms–3000 ms) were obtained for the T_1_ calculations, amounting to a total scan time of 12 minutes. Inversion recovery data from the imaging slice were acquired after selective inversion interleaved with nonselective inversion. Relative cerebral blood flow (CBF) was measured in the cortex, in the hippocampus, and in the thalamus based on [17].

Diffusion of water was imaged as described previously [35, 39]. In short, 22 axial slices covering the whole brain were acquired with a four-shot SE-EPI protocol. B0 shift compensation, navigator echoes, and an automatic correction algorithm to limit the occurrence of ghosts and artefacts were implemented. Encoding b-factors of 0 s/mm^2^ (b0 images; 5x) and 1000 s/mm^2^ were used and diffusion-sensitizing gradients were applied along 30 noncollinear directions in three-dimensional space.

The diffusion tensor was estimated for every voxel using the PATCH algorithm [40]; mean water diffusivity (MD) and fractional anisotropy (FA) were derived from the tensor estimation following a protocol as described elsewhere [35]. MD and FA values were measured in several white matter (WM) and gray matter (GM) areas, which were manually selected based on an anatomical atlas [41].

The resting-state fMRI (rsfMRI) datasets were first realigned using a least-squares method and rigid-body transformation with Statistical Parametric Mapping (SPM) mouse toolbox (SPM5, University College London; http://www.fil.ion.ucl.ac.uk/spm/; [42]). Mean and maximum displacement across the six degrees of freedom (along the *x*-, *y*-, and *z*-axes and on three rotation parameters pitch, roll, and yaw) were measured in each mouse. The mean SE-EPI images of each mouse were then used to generate a study-specific template through linear affine and nonlinear diffeomorphic transformation (ANTs. v1.9; http://picsl.upenn.edu/ANTS/). Visual inspection of the normalized dataset was performed to check for possible normalization biases. On the template, 15 areas were selected in left and right hemisphere. The selected regions were based on previous work in functional connectivity in mice [43] and included left and right dorsal hippocampus, left and right ventral hippocampus, left and right auditory cortex, left and right motor cortex, left and right somatosensory cortex, and left and right visual cortex. All cortical ROIs were selected 1-2 voxels away from the edge of the cortex, to minimize the impact of susceptibility-weighted artefacts, which are more prominent in areas of different tissues interface (e.g., near the skull or near the ear canals). After motion regression, in-plane spatial smoothing (0.4 × 0.4 mm), linear detrending, and temporal high-pass filtering (cutoff at 0.01 Hz) were applied to compensate for small across-mouse misregistration and temporal low-frequency noise. FC group comparison between ROIs was calculated from the BOLD time series using total correlation and partial correlation analyses implemented in FSLNets (FSLNets v0.3; http://fsl.fmrib.ox.ac.uk/fsl/fslwiki/). Pearson's correlation values were Fisher transformed to *Z*-scores for group comparisons and statistical analysis.

### 2.4. Brain Tissue Preparation

Directly following the MR measurements at 12 and 18 months of age, anaesthetised mice were sacrificed by transcardial perfusion with 0.1 M phosphate buffered saline (PBS). The perfused brains were cut sagittally and the right hemispheres were snap frozen in liquid nitrogen and stored at −80°C, before further biochemical processing. The left hemispheres were immersion fixated for 15 h at 4°C in 4% paraformaldehyde fixative and thereafter stored in 0.1 M PBS with 0.01% sodium azide at 4°C for immunohistochemical staining.

### 2.5. Immunohistochemistry: PSD95

Eight series of 30 *μ*m coronal sections were cut through the brain using a sliding microtome (Microm HM 440 E, Walldorf, Germany) equipped with an object table for freeze sectioning at −60°C. The tissue was stained for postsynaptic density with polyclonal rabbit anti-PSD95 antibody (1 : 2000; Abcam Cat # ab18258, RRID:AB_444362) using one complete series of brain sections. Immunohistochemistry was performed using standard free-floating labelling procedures, as described previously.

### 2.6. Quantification

The stained sections were analysed using a Zeiss Axioskop microscope equipped with hardware and software of Microbrightfield (Williston, VT, USA). Brain regions were based on the mouse brain atlas of Franklin and Paxinos (third edition, 2008) and quantified in five regions of the hippocampus: the inner molecular layer (IML), outer molecular layer (OML), cornus ammonis 1 (CA1), CA2, and CA3. Additionally, two regions in the cortex corresponding to the visual and somatosensory cortex were analysed. The relevant regions were digitized at 100x magnification with immersion oil using Stereo Investigator. The quantification of the photographs was performed using Image J (Image J, U.S. National Institutes of Health, Bethesda, Maryland, USA). The contrast was manually enhanced, following the same procedure for all digitized images, and the amount of tissue stained was measured with a threshold-based approach.

### 2.7. Biochemical Analyses

Serum and brain sterol levels were measured by gas-chromatography-mass-spectrometry-selected-ion-monitoring (GC-MS-SIM) as described in detail previously [44]. The cerebellum of the right hemisphere was homogenized and sterols were extracted overnight by chloroform/methanol trimethylsilylation prior to GC-MS-SIM analysis [44]. Brain fatty acid analyses were performed with a part of the brain homogenate (olfactory bulb and part of frontal cortex), as described previously [35].

### 2.8. Statistics

For the statistical analysis, IBM SPSS 20 software (IBM Corporation, New York, NY, USA) was used. Since the setup of the current study was designed to determine the effects of diet supply at two stages in which apoE4 mice may develop different neuropathological traits of AD, statistical analyses were performed separately for the two age-points.

Multivariate ANOVA (MANOVA) with Bonferroni corrections, using body weight as covariate when necessary, was conducted with between-group-factors genotype and diet to analyse possible differences in all the other parameters. If the Bonferroni* post hoc* test indicated a significant interaction between genotype and diet, the data were split for the concerning factor and thereafter analysed again with the MANOVA. Statistical significance was set at *p* ≤ 0.05. All data are expressed as mean ± SEM.

## 3. Results

Mortality rate in the apoE4 mice was normal until age of 18 months (<8% total mortality rate) in both diet groups. None of the WT animals died during the experiment.

### 3.1. Body Weight

**Figure 2:**
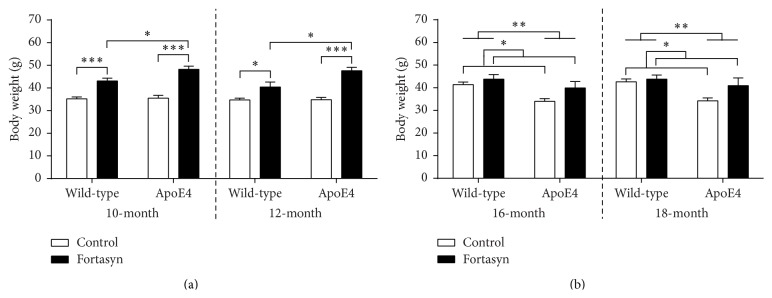
Body weight was measured in 10–12-month-old (a) and 16–18-month-old ApoE4 and wild-type (WT) mice. (a) At 10 months of age, ApoE4 and also wild-type mice on Fortasyn were significantly heavier than on control diet (ApoE4: *p* < 0.001; wild-type: *p* < 0.001). Only on Fortasyn diet was a genotype effect found showing a higher weight of ApoE4 mice compared with wild-type mice (*p* = 0.015). Again at 12 months of age, all animals on Fortasyn diet showed an increased body weight compared to animals on control diet (ApoE4: *p* < 0.001; wild-type: *p* = 0.019). ApoE4 mice on Fortasyn were heavier than wild-type mice on the same diet (*p* = 0.013). (b) At 16 and also 18 months of age, ApoE4 mice were significantly lighter than wild-type mice (16-month: *p* = 0.003; 18-month: *p* = 0.005). Furthermore, all animals on Fortasyn were heavier than animals on control diet (16-month: *p* = 0.024; 18-month: *p* = 0.042). ^*∗*^ *p* ≤ 0.05; ^*∗∗*^ *p* ≤ 0.01; ^*∗∗∗*^ *p* ≤ 0.001.

*10–12-Month-Old Mice*. Body weight was measured at 10 and 12 months of age (Figure 2(a)). Statistical analysis revealed a significant genotype × diet interaction (*p* = 0.050). ApoE4 and WT mice fed the Fortasyn diet were significantly heavier than animals on control diet (ApoE4: *F*(1,18) = 46.6, *p* < 0.001; WT: *F*(1,17) = 31.2, *p* < 0.001). ApoE4 mice on Fortasyn were significantly heavier compared to WT mice on Fortasyn (*F*(1,17) = 7.3, *p* = 0.015).

At the start of the MRI measurements a significant genotype × diet interaction on body weight was found (*p* = 0.018). Again, all animals on Fortasyn diet showed an increased body weight compared to animals on control diet (ApoE4: *F*(1,18) = 50.1, *p* < 0.001; wild-type: *F*(1,17) = 6.7, *p* = 0.019). ApoE4 mice on Fortasyn were heavier than wild-type mice on the same diet (*F*(1,17) = 7.6, *p* = 0.013).

*16–18-Month-Old Mice*. Body weight was measured at 16 and 18 months of age (Figure 2(b)). In both measurements, ApoE4 mice weighed significantly less than WT mice (16 months: *F*(1,33) = 10.3, *p* = 0.003; 18 months: *F*(1,33) = 9.0, *p* = 0.005). Both at 16 and at 18 months of age, all animals on Fortasyn were heavier than animals on control diet (16 months: *F*(1,33) = 5.6, *p* = 0.024; 18 months: *F*(1,33) = 4.5, *p* = 0.042).

### 3.2. Magnetic Resonance Imaging

#### 3.2.1. Cerebral Blood Flow

To study group-related differences on cerebrovascular health, we measured cerebral blood flow (CBF) with a flow-sensitive MRI technique (FAIR ASL). Three regions of interest (ROIs) on the left and right brain hemispheres were analysed: cortex, hippocampus, and thalamus. Since no intraindividual differences in CBF between right and left hemispheres were detected between mice groups (data not shown), values from both sides were averaged. In all measures, CBF was not significantly influenced by body weight.

**Figure 3:**
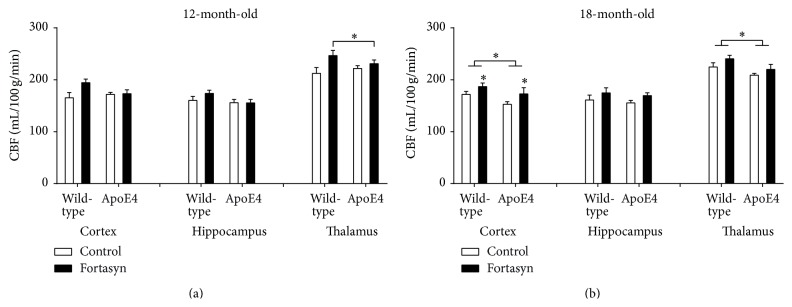
Cerebral blood flow (CBF) as measured with a flow-sensitive MRI technique (FAIR ASL) at 12 months and 18 months of age in C57BL/6J wild-type control mice and ApoE4 transgenic mice on control diet or Fortasyn diet. Three regions of interest (ROIs) on the left and right brain hemispheres were analysed: cortex, hippocampus, and thalamus. (a) In the 12-month-old mice we detected a genotype × diet interaction in the thalamus (*p* = 0.045). Fortasyn diet increased thalamic CBF (*p* = 0.040) more strongly in wild-type mice than in the apoE4 littermates. (b) In the 18-month-old mice, CBF was decreased in the cortex (*p* = 0.044) and in the thalamus (*p* = 0.023) of apoE4 mice as compared to wild-type mice. Cortical CBF was increased by the Fortasyn diet in both wild-type and apoE4 mice (*p* = 0.038). ^*∗*^ *p* ≤ 0.05; ^*∗∗*^ *p* ≤ 0.01; ^*∗∗∗*^ *p* ≤ 0.001.

In the 12-month-old mice, we detected a genotype × diet interaction in the thalamus (*p* = 0.045). In detail, Fortasyn diet increased thalamic CBF (*F*(1,16) = 5.0, *p* = 0.040) more strongly in WT mice than in the apoE4 littermates (Figure 3(a)).

In the 18-month-old mice (Figure 3(b)), CBF was decreased in the cortex (*F*(1,31) = 4.4, *p* = 0.044) and in the thalamus (*F*(1,31) = 5.7, *p* = 0.023) of apoE4 mice as compared to WT mice. Cortical CBF was increased by the Fortasyn diet in both WT and apoE4 mice (*F*(1,31) = 4.7, *p* = 0.038).

#### 3.2.2. Diffusion Tensor Imaging

**Figure 4:**
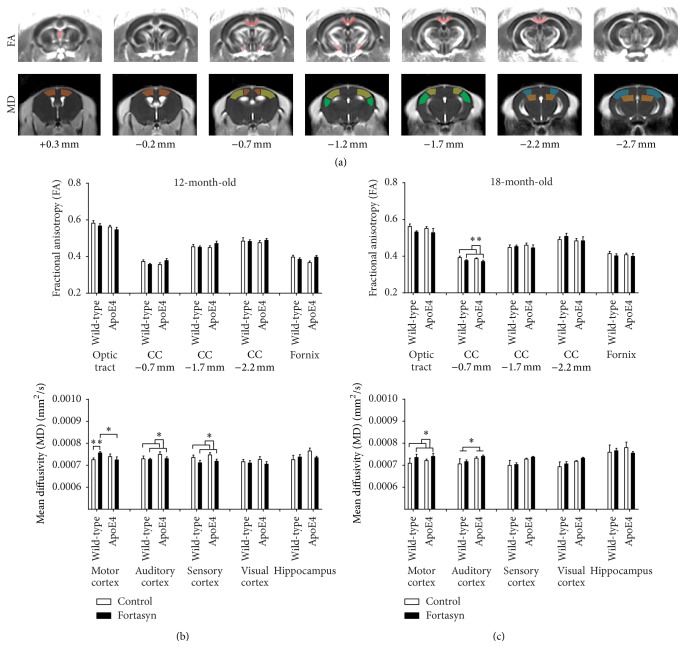
Quantitatively assessed diffusion tensor derived indices at 12 months and 18 months of age in C57BL/6J wild-type control mice and ApoE4 transgenic mice on control diet or Fortasyn diet. (a) Fractional anisotropy (FA) and mean diffusivity (MD) were measured for ROIs drawn in white and gray matter regions, respectively. (b) In 12-month-old mice, no differences in FA were observed. In the 12-month-old mice, the wild-type mice on Fortasyn diet had higher MD in the motor cortex than wild-type mice on control diet (*p* = 0.009). Furthermore apoE4 mice on Fortasyn diet had a lower MD in the motor cortex than wild-type mice on Fortasyn (*p* = 0.036). Fortasyn diet decreased MD in the auditory cortex (*p* = 0.034) and in the somatosensory cortex (*p* = 0.009) compared to control diet, irrespective of genotype. (c) At 18 months of age, Fortasyn fed mice had a lower FA at −0.7 mm in the corpus callosum than control fed mice (*p* = 0.008). At 18 months of age, apoE4 mice displayed an increased MD in the auditory cortex (*p* = 0.025). ^*∗*^ *p* ≤ 0.05; ^*∗∗*^ *p* ≤ 0.01; ^*∗∗∗*^ *p* ≤ 0.001.

Quantitative assessment of diffusion tensor derived indices was performed for ROIs drawn in white and gray matter regions to assess genotype and diet effects in apoE4 and nontransgenic WT mice (Figure 4(a)).

*Fractional Anisotropy*. In the 12-month-old mice, we did not detect any significant differences in white matter FA between the groups of mice (Figure 4(b)).

At 18 months of age (Figure 4(c)), Fortasyn fed mice showed a lower FA at −0.7 mm in the corpus callosum compared to control fed mice (*F*(1,31) = 4.7, *p* = 0.008).

*Mean Diffusivity*. In the 12-month-old mice, the MANOVA revealed a genotype × diet interaction for MD in the motor cortex (*p* = 0.024, Figure 4(b)).

In detail, WT mice on Fortasyn diet had higher MD in the motor cortex than wild-type mice on control diet (*F*(1,14) = 9.2, *p* = 0.009). Furthermore apoE4 mice on Fortasyn diet had a lower MD in the motor cortex than WT mice on Fortasyn (*F*(1,17) = 5.2, *p* = 0.036). Fortasyn diet decreased MD in the auditory cortex (*F*(1,29) = 4.9, *p* = 0.034) and in the somatosensory cortex (*F*(1,29) = 7.9, *p* = 0.009) compared to control diet, irrespective of genotype.

At 18 months of age (Figure 4(c)), apoE4 mice displayed an increased MD in the auditory cortex (*F*(1,29) = 5.6, *p* = 0.025) compared to WT mice.

#### 3.2.3. rsfMRI

**Figure 5:**
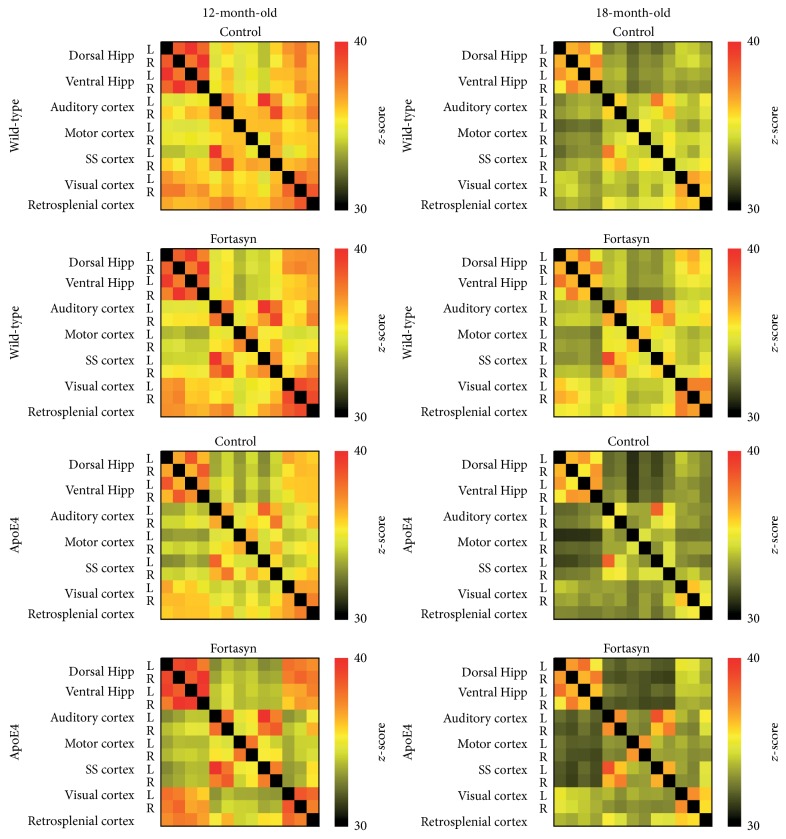
Resting-state functional connectivity (FC) based on total correlation analyses of 15 regions of interest (ROIs) in the mouse brain. Total correlation matrices of wild-type and apoE4 at 12 and 18 months of age, both on Fortasyn and control diets. At 12 months of age, multivariate ANOVA (MANOVA) revealed some significant genotype but no diet effects in apoE4 compared to wild-type mice. More specifically, apoE4 mice showed reduced FC between the right auditory cortex and the left dorsal hippocampus (*p* = 0.033) and also between the left visual cortex and the right auditory cortex (*p* = 0.028). At 18 months of age, apoE4 mice had significant lower FC between cortical and hippocampal regions but also between cortical regions themselves. Notably, Fortasyn was able to increase the hippocampal FC and also FC between the visual and retrosplenial cortices to the hippocampus.

**Figure 6:**
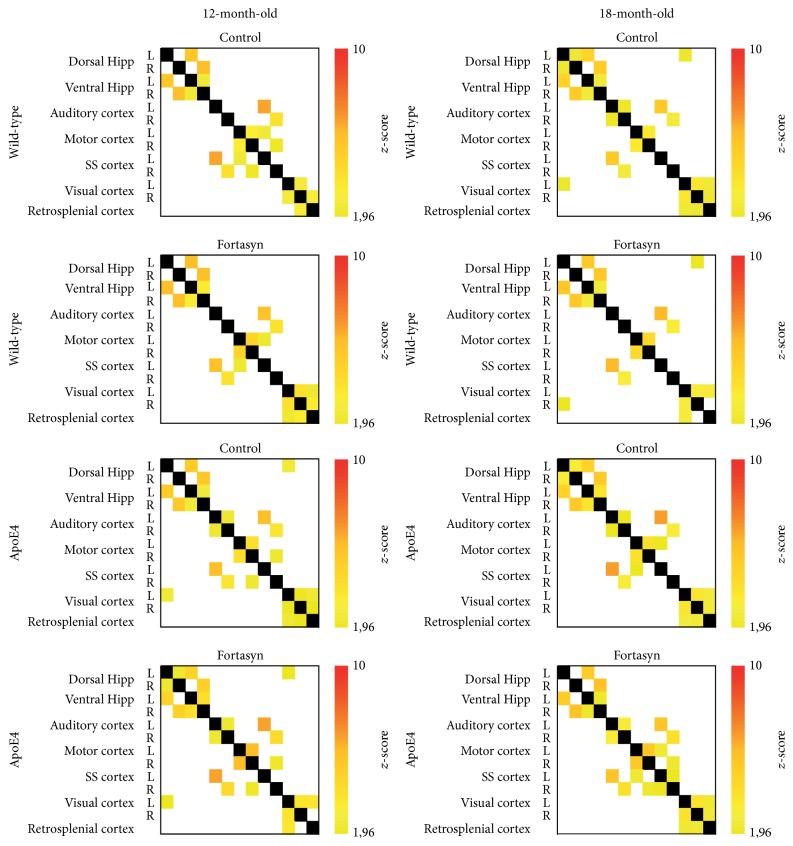
Resting-state functional connectivity (FC) based on partial correlation analyses of 15 regions of interest (ROIs) in the mouse brain. Total correlation matrices of wild-type and apoE4 at 12 and 18 months of age, both on Fortasyn and control diets. At 12 months of age, apoE4 mice showed a reduced FC pattern between left dorsal and ventral hippocampus (*p* = 0.040). Additionally, animals on Fortasyn compared to control diet showed an increased partial correlation in the interhemispheric connection between left and right ventral hippocampus (*p* = 0.012). At 18 months of age, ApoE4 mice on both diets had a reduced FC between the right auditory cortex and the right motor cortex (*p* < 0.001). The Fortasyn diet caused contrasting effects: a higher FC was found between left-to-right motor cortices (*p* = 0.013), but a slight FC reduction was revealed between left and right dorsal hippocampus (*p* = 0.045). Fortasyn diet increased partial correlation in apoE4 mice compared to their wild-type littermates between left and right auditory cortex (*p* = 0.015) and between right auditory cortex and right somatosensory cortex (*p* = 0.015). In wild-type mice FC between left somatosensory cortex and left auditory cortex (*p* = 0.041) was higher in Fortasyn fed animals compared to control fed animals.

*Total Correlation Analyses*. To compare the FC patterns between different genotypes and diets, rsfMRI data were statistically analysed based on total correlation (Figure 5) and partial correlation (Figure 6).

At 12 months of age, multivariate ANOVA (MANOVA) revealed some significant genotype but no diet effects in apoE4 compared to WT mice. More specifically, apoE4 mice showed reduced FC between the right auditory cortex and the left dorsal hippocampus (*F*(1,24) = 5.1, *p* = 0.033) and also between the left visual cortex and the right auditory cortex (*F*(1,24) = 5.5, *p* = 0.028).

At 18 months of age, the MANOVA demonstrated several significant genotype and diet effects. In detail, apoE4 mice had significant lower FC between cortical and hippocampal regions but also between cortical regions themselves. Notably, Fortasyn was able to increase the hippocampal FC and also FC between the visual and retrosplenial cortex to the hippocampus.

The MANOVA revealed genotype × diet interactions in the left auditory cortex to retrosplenial cortex (*p* = 0.011), in the right auditory cortex to right somatosensory cortex (*p* = 0.05), and in the left somatosensory cortex to retrosplenial cortex (*p* = 0.025). ApoE4 mice on control diet showed a reduced FC between left auditory cortex and retrosplenial cortex (*F*(1,15) = 17.9, *p* = 0.001), right auditory cortex and right somatosensory cortex (*F*(1,15) = 4.9, *p* = 0.042), and left somatosensory cortex and retrosplenial cortex (*F*(1,15) = 18.2, *p* = 0.001), which were not observed in ApoE4 mice on Fortasyn diet.

Moreover, compared to their transgenic littermates on control diet only apoE4 mice on Fortasyn displayed an increased FC between left auditory cortex and retrosplenial cortex (*F*(1,11) = 13.0, *p* = 0.004), right auditory cortex and right somatosensory cortex (*F*(1,11) = 9.9, *p* = 0.009), and left somatosensory cortex and retrosplenial cortex (*F*(1,11) = 10.2, *p* = 0.009).

*Partial Correlation Analyses*. At 12 months of age, significant genotype and diet effects were shown using MANOVA (Figure 6). In detail, apoE4 mice showed a reduced FC between left dorsal and ventral hippocampus (*F*(1,24) = 4.7, *p* = 0.040). Additionally, animals on Fortasyn diet showed an increased partial correlation in the interhemispheric connection between left and right ventral hippocampus (*F*(1,24) = 7.3, *p* = 0.012).

At 18 months of age, the MANOVA showed several significant genotype and diet effects (Figure 6). ApoE4 mice had a reduced FC between the right auditory cortex and the right motor cortex (*F*(1,26) = 16.4, *p* < 0.001). The Fortasyn diet increased FC between left and right motor cortices (*F*(1,26) = 7.1, *p* = 0.013) but slightly reduced FC between left and right dorsal hippocampus (*F*(1,26) = 4.4, *p* = 0.045).

MANOVA also revealed a genotype × diet interaction between right auditory cortex and right somatosensory cortex, *p* = 0.010. Fortasyn diet increased partial correlation in apoE4 mice compared to WT between right auditory cortex and right somatosensory cortex (*F*(1,12) = 8.1, *p* = 0.015).

### 3.3. Postsynaptic Density-95 (PSD-95) Protein

**Figure 7:**
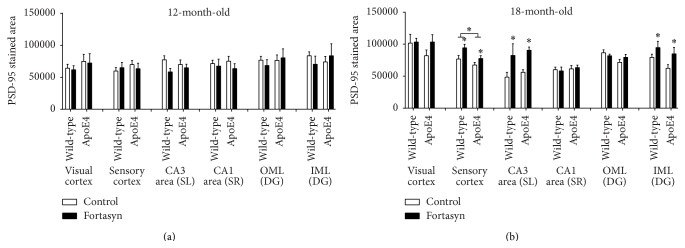
Levels of postsynaptic density-95 (PSD-95) in various brain areas in wild-type mice and apoE4 mice on either control diet or Fortasyn diet. (a) At 12 months, we did not find significant differences between groups. (b) At 18 months, reduced PSD-95 levels were seen in the sensory cortex of apoE4 mice compared to wild-type mice (*p* = 0.021). Fortasyn diet increased levels of PSD-95 in the sensory cortex (*p* = 0.014), CA3 area (*p* = 0.004), and IML (*p* = 0.004), irrespective of genotype. No genotype × diet interactions were observed. ^*∗*^ *p* ≤ 0.05; ^*∗∗*^ *p* ≤ 0.01; ^*∗∗∗*^ *p* ≤ 0.001.

Density of postsynaptic density proteins was visualised and quantified immunohistochemically in various cortical and hippocampal areas with polyclonal rabbit anti-PSD-95. At 12 months, we did not observe significant differences between groups (Figure 7). At 18 months, reduced PSD-95 levels were seen in the sensory cortex of apoE4 mice compared to WT mice (*F*(1,33) = 5.8, *p* < 0.021). Fortasyn diet increased levels of PSD-95 in the sensory cortex (*F*(1,33) = 6.7, *p* < 0.014), CA3 area (*F*(1,31) = 9.9, *p* < 0.004), and IML (*F*(1,31) = 9.9, *p* < 0.004), irrespective of genotype. No genotype × diet interactions were observed.

### 3.4. Fatty Acids

Fatty acid content was analysed in the brains of apoE4 and WT mice (Supplementary Material + Supplementary Table 1 available online at http://dx.doi.org/10.1155/2016/6846721). At both 12 and 18 months of age, increased relative levels of omega-3 fatty acids (*p* = 0.000) and an increased ratio of omega 3/6 were found in Fortasyn fed mice compared to their littermates on control diet (*p* = 0.000). At 18 months of age, ApoE4 mice displayed significantly increased relative arachidonic acid (*p* = 0.022) and relative omega-6 content (*p* = 0.038) compared to wild-type mice. For a detailed overview see the fatty acid section in the Supplementary Material and Supplementary Table 1.

### 3.5. Sterol Levels

Sterol levels were determined in the blood plasma (serum) and in the cerebellum of the brain. The main findings are described below; for all detailed results see Supplementary Material and Supplementary Tables 2 and 3, respectively.

#### 3.5.1. Blood Serum

At 12 months of age, apoE4 mice displayed increased level of the cholesterol precursor, dihydrolanosterol (*p* = 0.000), compared to WT mice. At 12 months of age, apoE4 mice on control diet had higher levels of lathosterol (*p* = 0.002) and lanosterol (*p* = 0.003) compared to WT mice on control diet.

At 18 months of age, apoE4 and WT mice on control diet displayed increased levels of campesterol (*p* = 0.000), sitosterol (*p* = 0.016), lanosterol (*p* = 0.010), desmosterol (*p* = 0.000), 24OH-cholesterol (*p* = 0.002), and cholesterol (*p* = 0.033) compared to apoE4 and WT mice on Fortasyn diet.

#### 3.5.2. Cerebellum

At 12 months of age, apoE4 and WT mice on control diet demonstrated increased levels of lathosterol (*p* = 0.003), campesterol (*p* < 0.000), and lanosterol (*p* = 0.001) in the cerebellum.

At 18 months of age, cerebellar cholesterol levels were unchanged in ApoE4 mice compared to WT mice (*p* = 0.614). In control fed mice levels of campesterol and precursors of cholesterol, lathosterol, and lanosterol were higher than in Fortasyn fed mice (campesterol, *p* < 0.000; lathosterol, *p* < 0.000; lanosterol, *p* = 0.011).

## 4. Discussion

### 4.1. ApoE4 Mice as Model for the Early Asymptomatic Phase in AD

In this study, we investigated the extent to which apoE4 mice display cerebrovascular flaws, synaptic loss, and connectivity during aging. The *ε*4 allele of the apoE gene is strongly associated with sporadic AD [45]. Among several proposed mechanisms by which apoE4 promotes AD, there are indications that apoE4 is less effective in synaptic repair and remodelling processes compared to other isoforms [46, 47]. Moreover, apoE4 carriers are clearly more susceptible to vascular brain damage (e.g., stroke, brain haemorrhage [29, 30, 48]), and they display aberrant functional connectivity [49].

Similarly, the apoE4 mouse line exhibits increased risk of developing vascular disorders and neuronal deficits due to altered cholesterol metabolism, especially when challenged with a high-fat diet [33]. Recently we also described spontaneous functional connectivity deficits in these mice, possibly associated with cerebral blood flow reduction [50]. Because these deficits seem to be aggravated with aging we investigated this mouse line at two different ages.

At 10–12 months of age, apoE4 mice did not display many differences compared to WT animals. Despite some slight alterations in the sterol levels of 12-month-old apoE4 mice, all other measured parameters including cerebral blood flow and number of postsynapses were unaffected compared to WT animals. Previously, we have shown that at this age also cerebral blood volume, amount of presynaptic boutons, and neurogenesis did not differ from WT mice [35]. The lack of cerebrovascular alterations like a reduced CBF at this age may explain the absence of pathologies.

However at 16–18 months of age, apoE4 mice revealed reduced CBF and accelerated neurodegeneration, which are typical features of prodromal AD [51–54]. Specifically, we detected reduced thalamic and cortical perfusion, reduced cortical postsynaptic density, increased cortical mean diffusivity (MD), and reduced fractional anisotropy (FA) in white matter tracts. Similar changes in brain diffusivity, as a biomarker for white and gray matter integrity, have been reported in human apoE4 carriers [55–58]. These structural modifications may be linked to the isoform-specific role of apoE in synaptic development, dendrite formation, and axonal guidance, which to some extent may be impaired in apoE4 carriers [59]. Nevertheless, these results were not consistent across different ages, suggesting that changes in WM/GM microstructure properties may not directly reflect an associated AD-like pathology [60]. Moreover, at this age we measured a widespread reduction in functional connectivity at rest, which was previously reported [17].

Overall, in line with other studies in this mouse model [34], these findings suggest that the apoE4 mice spontaneously develop age- and apoE4-dependent accelerated brain pathology. This is in agreement with human studies on apoE4 carriers showing a faster decline of CBF during aging [61, 62] and reduced connectivity between cortical regions at old age [63–65]. However, the apoE4 mice exhibited relatively small genotype effects like CBF decline and reduced connectivity just at 18 months of age and not at 12 months of age. Therefore, one could argue that an 18-month-old apoE4 mouse is at most similar to a 65-year-old still healthy, possibly only mildly impaired human apoE4 carrier, showing no signs of dementia yet and carrying no multiple risk factors and comorbidities. It has been found, namely, that human apoE4 carriers often carry multiple risk factors (genetic modifiers, comorbidities, and lifestyle factors) that contribute significantly to synaptic integrity. The only risk factor included in our mice was age, combined with apoE4 genotype causing mild pathology.

A recent review on apoE-related biomarker profiles in the early phase of AD further elucidates this relatively novel concept of the apoE4 to be considered more as a vulnerability factor rather than a pathogenic factor [66]. Based on this idea, it is the interaction between these vulnerabilities and the age-related pathological events that may trigger synaptic loss, contributing to the development of AD. In our study, this aging-dependency of apoE4 seemed confirmed, as most of the biomarkers for brain deficits were identified only in the 18-month-old apoE4 group, representing an early stage of the disease, when complete early AD-like pathology is still not fully developed. Holding this hypothesis, the model becomes extremely attractive to study the effects of nutritional intervention as a preventive strategy against early AD-like pathology.

### 4.2. Dietary Intervention

In the current experiment we fed the mice a specific multinutrient supplementation diet designed to ameliorate synapse loss and to reduce membrane-related pathology in AD by providing nutritional precursors and cofactors to support neuronal membrane formation and function [67]. This nutrient combination, called Fortasyn, comprises uridine, docosahexaenoic acid, eicosapentaenoic acid, choline, phospholipids, folic acid, vitamins B12, B6, C, and E, and selenium. Some of the components in these diets, such as omega-3 long-chain polyunsaturated fatty acids (n3- LCPUFAs), have also been shown to improve vascular health [5, 68–71]. The results confirmed our initial hypothesis that the Fortasyn diet has the potential to reduce the occurrence of vulnerabilities for AD by simultaneously improving cerebrovascular health and enhancing neuroprotective mechanisms. However it is also possible that the capacity of the diet to support membrane phospholipid synthesis could underlie both the synapse formation/functional connectivity and the effects on cerebrovascular functioning. Besides, the combination of phosphatide precursors like n3-LCPUFAs, uridine, and choline has proven to synergistically increase the synthesis of synaptic proteins and phospholipids in the brain [14–16].

These findings confirm novel important mechanisms by which these diets may affect AD onset and development, similar to earlier findings in a transgenic AD mouse model [17]. Particularly, among the different parameters analysed, the strongest and most consistent dietary effect in these studies involves the improvement of cerebrovascular health and functional connectivity. Several epidemiological studies and controlled trials showed a correlation between B vitamins, n3-LCPUFAs, and MUFA (like oleic acid from olive oil and nuts) and improvements in autonomic function, lowered blood pressure, reduced atherosclerosis, reduced total homocysteine, and enhanced microvascular endothelium-dependent vasodilation processes [4, 72, 73].

All these factors may contribute to an improved functionality of the brain vasculature, with a beneficial effect particularly in apoE4 mice, in which these pathologies are aggravated. Interestingly, increased cortical CBF and levels of postsynaptic markers were found in both apoE4 and WT animals on Fortasyn diet; these findings suggest that the diet had a similar effect in both genotypes and its contribution is not determined by the apoE genotype. We have also shown that the Fortasyn diet affected brain fatty acid profiles in both genotypes (Supplementary Material, Table 1), by decreasing the relative concentration of n6-fatty acids (notably arachidonic acid), which is significantly increased in the 18-month-old apoE4 mice compared to WT, and by increasing the relative concentration of n3-fatty acids (notably DHA) and monounsaturated fatty acids, especially oleic acid. These supplementary results indicate a replacement of n6 fatty acids from cell membranes in favour of n3-LCPUFAs (reflected by the increased n3/n6 ratio), which has beneficial effects on membrane fluidity, and neuronal transmission and signalling [74–76]. Furthermore, we have shown that the Fortasyn diet similarly affected serum (Supplementary Material Table 2) and brain sterol level (Supplementary Material Table 3). In detail, in the plasma of aged animals on Fortasyn we found decreased levels of cholesterol but also of precursors like lanosterol and derivates of cholesterol like 24OH-cholesterol. Notably, in the cerebellum of aged animals on Fortasyn we detected a decreased concentration of the cholesterol precursor lanosterol and increased level of a derivate of cholesterol, 24S-hydroxycholesterol. In the brain, the enzyme, cholesterol 24S-hydroxylase, converts cholesterol to 24S-hydroxycholesterol. This mechanism is the most important pathway for the elimination of brain cholesterol and the maintenance of brain cholesterol homeostasis [77–80]. These results are also in line with our previous study using another AD mouse model overexpressing A*β*. Here, another cholesterol precursor, lathosterol, was decreased in the brain of Fortasyn fed AD and WT mice, while again an increased cerebral level of the derivate of cholesterol, 24S-hydroxycholesterol, was measured in Fortasyn fed AD and WT mice [81]. Our data indicate a higher elimination rate of brain cholesterol in Fortasyn fed mice.

This may also explain the improved white matter integrity and preserved functional connectivity in both WT and AD mice fed with Fortasyn. In support of this hypothesis, it has been demonstrated that animals fed with a diet containing uridine monophosphate (UMP), n3-LCPUFAs, and choline showed increased levels of brain phospholipids, dendritic spines, and neurite outgrowth [14–16].

## 5. Conclusions

Overall, the study presented here further proved that two simultaneous protective mechanisms on vascular and synapse health are both enhanced by the specific Fortasyn diet and may strengthen each other synergistically, independent of the apoE genotype. The beneficial effect of these diets is suggested to be caused by increased production of phospholipids to sustain synaptic genesis and repair processes [14, 82]. In this and other recent experiments, we showed that a strong effect of these diets also involves the amelioration of cerebrovascular health. Although decreased cerebral perfusion has been recognized as an early and important contributor to AD pathology and cognitive decline [83], we believe that this aspect is not sufficiently considered in human nutritional intervention studies. In our mice, we have detected most of the pathological effects of apoE4 just at 18 months of age, and therefore, most of the beneficial effects of the diet were just present at 18 months of age. It is important to stress that our apoE4 mouse model only represents susceptibility to cognitive impairment and just like in human apoE4 carriers multiple risk factors are required in combination with apoE4 to precipitate disease pathology. For future research it would therefore be interesting to study the effect of the Fortasyn diet in older (24 months of age) apoE4 mice, in apoE4 mice with induced apoE4 comorbidities like hypertension, stroke, or in apoE4 mice on high-fat diet or in female apoE4 mice, resembling more closely the human susceptibility to AD. For example, in clinical studies on brain atrophy and clinical decline among cognitively normal older individuals and individuals with mild cognitive impairment and Alzheimer's disease it has been shown that the presence of apoE4 significantly accelerated rates of decline, and women in all cohorts had higher rates of decline than men [84]. Additionally, a preclinical study revealed that expression of human apoE4 renders aged mice fed with a western-type diet more susceptible to sensorimotor deficits upon stroke indicating an altered functional outcome following stroke in apoE4 carriers [85]. Furthermore, this study demonstrated the value of a multimodal approach, including advanced MR neuroimaging tools, for detecting changes in brain structure and function with respect to dietary intervention.

## Supplementary Material

Fatty acid content was determined in the olfactory bulb and part of frontal cortex (supplementary table 1). Sterol levels were determined in the blood plasma (serum) (supplementary table 2) and in the cerebellum of the brain (supplementary table 3).

## Figures and Tables

**Table 1:** Compositions of the experimental diets used, based on AIN-93M [37] with minor revisions.

Source	Dietary groups	
	Control [g/100 g]	Fortasyn [g/100 g]
Corn starch	35.57	33.12
Casein	14.00	14.00
Corn dextrin	15.5	15.50
Sucrose	10.00	10.00
Dextrose	10.00	10.00
Fibers	5.00	5.00
Mineral mix (AIN-93 M-MX)	3.50	3.50
Vitamin mix (AIN-93-VX)	1.00	1.00
*Fats*		
Soy oil	1.900	—
Coconut oil	0.900	0.100
Corn oil	2.200	1.870
Fish oil	—	3.030
*Additions*		
l-Cysteine	0.180	0.180
Choline bitartrate (41.1% choline)	0.250	0.250
tert-Butylhydroquinone	0.0008	0.0008
Pyridoxine–HCL	—	0.00328
Folic acid (90%)	—	0.00067
Cyanocobalamin (0.1% in mannitol)	—	0.00350
Ascorbic acid (100% pure)	—	0.160
dl-*α*-Tocopheryl acetate (500 IU/g)	—	0.4650
UMP disodium (24% H_2_O)	—	1.0
Choline chloride (74.576%)	—	0.402
Soy lecithin	—	0.402
Sodium selenite (46% min)	—	0.00023
Energy (kcal/100 g chow)	376.9	367.1

## Abbreviations

AD: Alzheimer's disease
CBF: Cerebral blood flow
FC: Functional connectivity
WT: Wild-type
n3-LCPUFAs: Omega-3 long-chain polyunsaturated fatty acids
DHA: Docosahexaenoic acid
EPA: Eicosapentaenoic acid
apoE: Apolipoprotein
A*β*: Amyloid-*β*
ES: Embryonic stem
GE: Gradient echo
MD: Mean water diffusivity
FA: Fractional anisotropy
PBS: Phosphate buffered saline
IML: Inner molecular layer
OML: Outer molecular layer
CA: Cornus ammonis
GC-MS-SIM: Gas-chromatography-mass-spectrometry-selected-ion-monitoring.
