# Supplementary material for: A Dietary Treatment Improves Cerebral Blood Flow and Brain Connectivity in Aging apoE4 Mice
Source: Neural Plast. 2016 Mar 10;2016:6846721. doi: 10.1155/2016/6846721 (PMC4806294; doi:10.1155/2016/6846721)
Supplement: Supplementary file 1 — Fatty acid content was determined in the olfactory bulb and part of frontal cortex (supplementary table 1). Sterol levels were determined in the blood plasma (serum) (supplementary table 2) and in the cerebellum of the brain (supplementary table 3). [file 6846721.f1.docx]

Supplementary material

**1 Fatty acids**

Fatty acid content was determined in the olfactory bulb and part of frontal cortex (supplementary table 1).

**12 months**

Overall diet effects in apoE4 and WT mice were observed for oleic acid (F(1,34)=56.1, *p*<0.001), mono-unsaturated fatty acids (MUFA; F(1,34)=8.9, *p*=0.005), arachidonic acid (F(1,34)=1164.2, *p*<0.001), and omega-6 fatty acids (F(1,34)=684.0, *p*<0.001). Compared to animals on control diet, apoE4 and WT mice on Fortasyn diet displayed increased relative oleic acid content, increased MUFA content, decreased arachidonic acid content, and decreased omega-6 fatty acid content (supplementary table 1). Significant genotype × diet interactions were observed for docosahexaenoic acid (DHA; *p*=0.037), omega-3 fatty acids (*p*=0.047) and omega3/6 ratio (*p*=0.047): both WT and apoE4 mice on Fortasyn diet displayed increased relative levels of docosahexaenoic acid (WT, F(1,17)=140.8, *p*<0.001; ApoE4, F(1,17)=83.4, *p*<0.001) and omega-3 fatty acids (WT, F(1,17)=178.2, *p*<0.001; ApoE4, F(1,17)=126.3, *p*<0.001), and an increased ratio of omega 3/6 compared to their littermates on control diet (WT, F(1,17)=326.0, *p*<0.001; ApoE4, F(1,17)=284.6, *p*<0.001). No significant genotype effects were found for these three parameters (supplementary table 1).

**18 months**

ApoE4 mice displayed significantly increased relative arachidonic acid (F(1,35)=5.7, *p*=0.022) and relative omega-6 content (F(1,35)=4.7, *p*=0.038) compared to WT mice. Overall diet effects were observed for oleic acid (F(1,35)=44.9, *p*<0.001), MUFA acids (F(1,35)=5.2, *p*=0.029), arachidonic acid (F(1,35)=1100.7, *p*<0.001), omega-6 fatty acids (F(1,35)=1155.0, *p*<0.001), DHA (F(1,35)=272.7, *p*<0.001), omega-3 fatty acids (F(1,35)=396.6, *p*<0.001) and omega-3/omega-6 ratio (F(1,35)=816.1, *p*<0.001). Compared to apoE4 and WT mice on control diet, apoE4 and WT mice on Fortasyn diet displayed increased relative oleic acid content, increased MUFA content, decreased arachidonic acid content, decreased omega-6 content, increased DHA content, increased omega-3 content and increased omega-3/omega-6 ratio (supplementary table 1).

**2 Sterol levels**

Sterol levels were determined in the blood plasma (serum) (supplementary table 2) and in the cerebellum of the brain (supplementary table 3).

**Blood serum**

At **12 months of age**, apoE4 mice displayed increased levels of stigmasterol (F(1,35)=16.3, *p*<0.001), brassicasterol (F(1,35)=17.9, *p*<0.001), dihydro-lanosterol (F(1,35)=26.7, *p*<0.001), and slightly, but non-significantly, decreased levels of cholesterol (F(1,35)=3.7, *p*=0.063) compared to WT mice. Overall diet effects were observed for campesterol (F(1,35)=12.8, *p*=0.001), stigmasterol (F(1,35)=51.2, *p*<0.001), brassicasterol (F(1,35)=73.4, *p*<0.001), dihydro-lanosterol (F(1,35)=4.3, *p*=0.046), and 24OH-cholesterol (*p*=0.001). Compared to animals fed a control diet, apoE4 and WT mice on Fortasyn diet displayed decreased levels of campesterol, brassicasterol, dihydro-lanosterol, and 24OH-cholesterol, while all mice on Fortasyn exhibited an increased level of stigmasterol compared to mice on control diet. Genotype diet interactions were found for desmosterol (*p*=0.059), lathosterol (*p*=0.014) and lanosterol (*p*=0.040). ApoE4 mice on Fortasyn diet displayed decreased levels of lathosterol (F(1,18)=7.8, *p*=0.012) and lanosterol (F(1,18)=9.4, *p*=0.007), and desmosterol (F(1,18)=34.0, *p*<0.001) compared to apoE4 mice on control diet, but increased levels of lathosterol (F(1,17)=9.8, *p*=0.006) and lanosterol (F(1,17)=8.9, *p*=0.008) compared to WT mice on Fortasyn diet. Furthermore, apoE4 mice on control diet had higher levels of lathosterol (F(1,17)=12.9, *p*=0.002) and lanosterol (F(1,17)=11.8, *p*=0.003) compared to WT mice on control diet.

At **18 months of age**, apoE4 mice displayed decreased levels of cholestanol (F(1,28)=21.5, *p*<0.001), campesterol (F(1,28)=14.6, *p*=0.001), stigmasterol (F(1,28)=5.2, *p*=0.031), sitosterol (F(1,28)=15.3, *p*=0.001), sitostanol (F(1,28)=8.1, *p*=0.008), avenasterol (F(1,28)=20.7, *p*<0.001), lanosterol (F(1,28)=3.5, *p*=0.074), desmosterol (F(1,28)=10.0, *p*=0.004), dihydro-lanosterol (F(1,28)=6.4, *p*=0.017), 24OH-cholesterol (F(1,28)=19.8, *p*<0.001), 7αOH-cholesterol (F(1,28)=10.3, *p*=0.003) and cholesterol (F(1,28)=42.3, *p*<0.001) compared to WT mice. Overall diet effects were found for lathosterol (F(1,28)=3.3, *p*=0.079) campesterol (F(1,28)=57.2, *p*<0.001), stigmasterol (F(1,28)=39.6, *p*<0.001), sitosterol (F(1,28)=6.5, *p*=0.016), avenasterol (F(1,28)=4.9, *p*=0.034), brassicasterol (F(1,28)=96.1, *p*<0.001), lanosterol (F(1,28)=7.7, *p*=0.010), desmosterol (F(1,28)=22.1, *p*<0.001), 24OH-cholesterol (F(1,28)=12.1, *p*=0.002) and cholesterol (F(1,28)=5.0, *p*=0.033). Compared to animals fed a control diet, apoE4 and WT mice on Fortasyn diet displayed decreased levels of campesterol, sitosterol, lanosterol, desmosterol, 24OH-cholesterol and cholesterol and increased levels of stigmasterol, avenasterol and brassicasterol.

**Cerebellum**

In brain tissue of 12-month-old mice, levels of desmosterol (F(1,35)=3.9, *p*=0.055), 24OH-cholesterol (F(1,35)=5.0, *p*=0.031), and 27OH-cholesterol (F(1,35)=6.2, *p*=0.018) were increased in apoE4 mice compared to WT mice. Overall diet effects were found for lathosterol (F(1,35)=10.5, *p*=0.003), campesterol (F(1,35)=37.2, *p*<0.001), lanosterol (F(1,35)=13.1, *p*<0.001), and stigmasterin (F(1,35)=20.7, *p*<0.001). Compared to animals fed a control diet, apoE4 and WT mice on Fortasyn diet displayed decreased levels of lathosterol, campesterol, and lanosterol, while levels of stigmasterin were increased in animals on Fortasyn diet compared to animals on control diet.

At 18 months of age, cerebellar cholesterol levels were unchanged in ApoE4 mice compared to WT mice (F(1,35)=0.3, *p*=0.614). Levels of lathosterol, campesterol, sitosterol, lanosterol and dihydrolanosterol were all decreased in ApoE4 mice compared to WT mice (Lathosterol, F(1,35)=8.2, *p*=0.007; Campesterol, F(1,35)=10.4, *p*=0.003; Sitosterol, F(1,35)=3.9, *p*=0.055; Lanosterol, F(1,35)=15.7, *p*<0.001; Dihydrolanosterol, F(1,35)=7.9, *p*=0.008). In Fortasyn fed mice lathosterol, campesterol and lanosterol were lower than in control fed mice (Lathosterol, F(1,35)=14.9, *p*<0.001; Campesterol, F(1,35)=65.0, *p*<0.001; Lanosterol, F(1,35)=7.3, *p*=0.011), while 24S-hydroxycholesterol was higher in these animals (F(1,35)=4.0, *p*=0.054).

**Table 1 Relative brain fatty acid content represented in average ± SEM for each experimental group. (Dark grey background + white letters: Diet-effect; Bright grey background + black letters: Genotype-effect; Black background + white letters: Genotype- + diet-effect). (Used abbreviations: PA=Palmitic acid; SA=Stearic acid; SFA=Saturated fatty acid; OA=Oleic acid; MUFA=Mono-unsaturated fatty acid; AA=Arachidonic acid; Ω-6=Omega-6; DHA=Docosahexaenoic acid; Ω-3=Omega-3; Ω 3/6=Omega 3/6)**

| **Brain fatty acid content** | | | | | | | | | | | | |
| --- | --- | --- | --- | --- | --- | --- | --- | --- | --- | --- | --- | --- |
|  |  |  | **PA** | **SA** | **SFA** | **OA** | **MUFA** | **AA** | **Ω-6** | **DHA** | **Ω-3** | **Ω 3/6** |
| **Age** | **Genotype** | **Diet** | **Relative fatty acid content (%)** | | | | | | | | | **Ratio** |
| 12 month | APO E4 | Fortasyn | 23,9±0,2 | 18,9±0,3 | 46,8±0,1 | ***15,1±0,1*** | ***21,8±0,2*** | ***7,5±0,1*** | ***10,6±0,1*** | ***19,4±0,2*** | ***20,7±0,2*** | ***1,9±0,0*** |
| 12 month | APO E4 | Control | 23,9±0,2 | 19,1±0,1 | 46,8±0,1 | 14,2±0,1 | 21,1±0,2 | 10,0±0,1 | 14,5±0,1 | 16,9±0,2 | 17,5±0,2 | 1,2±0,0 |
| 12 month | WT | Fortasyn | 24,1±0,2 | 18,9±0,3 | 46,8±0,1 | ***14,9±0,2*** | ***21,6±0,3*** | ***7,3±0,1*** | ***10,4±0,1*** | ***19,8±0,2*** | ***21,1±0,2*** | ***2,0±0,0*** |
| 12 month | WT | Control | 24,0±0,3 | 19,2±0,2 | 46,9±0,2 | 14,0±0,1 | 21,1±0,1 | 10,0±0,1 | 14,8±0,2 | 16,5±0,2 | 17,2±0,2 | 1,2±0,0 |
|  |  |  |  |  |  |  |  |  |  |  |  |  |
| 18 month | APO E4 | Fortasyn | 24,2±0,1 | 18,2±0,3 | 46,5±0,1 | ***15,0±0,1*** | ***21,7±0,2*** | ***7,4±0,1*** | ***10,5±0,1*** | ***20,0±0,2*** | ***21,3±0,2*** | ***2,0±0,0*** |
| 18 month | APO E4 | Control | 24,0±0,2 | 18,3±0,2 | 46,4±0,1 | 14,4±0,1 | 21,5±0,1 | ***9,8±0,1*** | ***14,2±0,1*** | 17,2±0,2 | 17,9±0,2 | 1,3±0,0 |
| 18 month | WT | Fortasyn | 24,2±0,3 | 18,1±0,3 | 46,5±0,1 | ***15,0±0,1*** | ***21,9±0,2*** | ***7,2±0,1*** | ***10,2±0,1*** | ***19,9±0,2*** | ***21,3±0,2*** | ***2,1±0,0*** |
| 18 month | WT | Control | 24,3±0,2 | 18,1±0,2 | 46,5±0,1 | 14,2±0,1 | 21,4±0,1 | 9,6±0,0 | 14,0±0,1 | 17,3±0,1 | 18,1±0,1 | 1,3±0,0 |

Table 2 Plasma sterol levels represented in average ± SEM for each experimental group. (Dark grey background + white letters: Diet-effect; Bright grey background + black letters: Genotype-effect; Black background + white letters: Genotype- + diet-effect)

| **Plasma sterol levels** | | | | | | | | | | | | | | | | | | |
| --- | --- | --- | --- | --- | --- | --- | --- | --- | --- | --- | --- | --- | --- | --- | --- | --- | --- | --- |
|  |  |  | **Cholestanol** | **Lathosterol** | **Campesterol** | **Campestanol** | **Stigmasterol** | **Sitosterol** | **Sitostanol** | **Avenasterol** | **Brassicasterol** | **Lanosterol** | **Desmosterol** | **Dihydro** | **24OH** | **7aOH** | **27OH** | **Cholesterol** |
|  |  |  |  |  |  |  |  |  |  |  |  |  |  | **-Lanosterol** | **Cholesterol** | **Cholesterol** | **Cholesterol** | **(GC)** |
| **Age** | **Genotype** | **Diet** | **[mg/dl]** | **[mg/dl]** | **[mg/dl]** | **[µg/dl]** | **[µg/dl]** | **[mg/dl]** | **[µg/dl]** | **1000*Ratio(EPI)** | **[µg/dl]** | **[µg/dl]** | **[mg/dl]** | **[µg/dl]** | **[ng/ml]** | **[ng/ml]** | **[ng/ml]** | **[mg/dl]** |
| 12 month | APO E4 | Fortasyn | 1,1±0,0 | ***0,0±0,0*** | 1,5±0,1 | 39,6±3,3 | ***43,0±3,5*** | 0,9±0,1 | 12,6±1,2 | 2,5±0,2 | ***4,2±0,4*** | ***8,7±1,0*** | ***0,1±0,0*** | ***1,3±0,1*** | 28,2±1,1 | 8,5±1,4 | 77,9±3,3 | ***95,8±3,1*** |
| 12 month | APO E4 | Control | 1,0±0,1 | ***0,1±0,0*** | 2,1±0,2 | 28,1±2,6 | ***23,0±1,9*** | 0,9±0,1 | 12,3±0,9 | 2,4±0,2 | ***12,3±0,9*** | ***17,8±2,8*** | 0,1±0,0 | ***2,2±0,5*** | 37,6±2,1 | 6,5±0,9 | 82,3±4,5 | ***107,3±3,6*** |
| 12 month | WT | Fortasyn | 1,3±0,2 | 0,0±0,0 | 1,1±0,2 | 36,4±4,0 | 30,9±2,8 | 0,8±0,1 | 10,4±1,1 | 2,4±0,3 | 2,1±0,3 | 5,3±0,6 | 0,1±0,0 | 0,4±0,0 | 29,8±1,7 | 10,4±3,9 | 79,8±10,1 | 124,3±5,9 |
| 12 month | WT | Control | 1,1±0,2 | 0,0±0,0 | 2,3±0,4 | 33,7±6,0 | 14,8±1,3 | 0,9±0,1 | 12,0±1,3 | 2,4±0,4 | 7,7±1,2 | 7,0±1,5 | 0,1±0,0 | 0,5±0,1 | 35,5±3,0 | 13,1±3,0 | 69,6±6,7 | 113,6±16,2 |
|  |  |  |  |  |  |  |  |  |  |  |  |  |  |  |  |  |  |  |
| 18 month | APO E4 | Fortasyn | ***0,8±0,1*** | ***0,0±0,0*** | ***1,3±0,2*** | 30,8±4,9 | ***31,8±4,7*** | ***0,7±0,2*** | ***9,4±1,4*** | ***2,0±0,4*** | ***3,1±0,4*** | ***9,7±1,0*** | ***0,1±0,0*** | ***0,6±0,1*** | ***28,5±1,7*** | ***3,2±1,0*** | 74,7±5,1 | ***92,2±8,2*** |
| 18 month | APO E4 | Control | ***1,0±0,1*** | 0,0±0,0 | ***2,7±0,2*** | 81,0±49,6 | ***16,7±1,5*** | ***1,0±0,1*** | ***11,6±0,6*** | ***2,6±0,2*** | 10,8±0,7 | ***13,9±1,4*** | ***0,1±0,0*** | ***0,9±0,1*** | ***35,6±1,9*** | ***5,2±2,1*** | 66,8±3,0 | ***100,5±6,8*** |
| 18 month | WT | Fortasyn | 1,8±0,3 | ***0,0±0,0*** | ***1,9±0,2*** | 50,0±5,7 | ***42,7±3,8*** | ***1,2±0,1*** | 13,6±1,5 | ***3,3±0,3*** | ***3,4±0,4*** | ***11,0±1,0*** | ***0,1±0,0*** | ***1,0±0,2*** | ***37,8±2,3*** | 13,8±2,8 | 68,0±4,9 | ***145,8±11,4*** |
| 18 month | WT | Control | 2,2±0,4 | 0,0±0,0 | 3,5±0,2 | 56,4±7,5 | 19,7±1,9 | 1,4±0,1 | 16,5±2,7 | 4,2±0,4 | 10,6±1,1 | 24,6±6,6 | 0,1±0,0 | 1,4±0,2 | 46,3±2,2 | 14,1±4,5 | 77,5±6,5 | 185,2±13,3 |

Table 3 Brain sterol levels represented in average ± SEM for each experimental group. (Dark grey background + white letters: Diet-effect; Bright grey background + black letters: Genotype-effect; Black background + white letters: Genotype- + diet-effect)

| **Brain sterol levels** | | | | | | | | | | | | | |
| --- | --- | --- | --- | --- | --- | --- | --- | --- | --- | --- | --- | --- | --- |
|  |  |  | **Cholestanol** | **Lathosterol** | **Campesterol** | **Stigmasterin** | **Sitosterol** | **Lanosterol** | **Dihydro** | **Desmosterol** | **24OH** | **27OH** | **Cholesterol** |
|  |  |  |  |  |  |  |  |  | **-Lanosterol** |  | **Cholesterol** | **Cholesterol** | **(GC)** |
| **Age** | **Genotype** | **Diet** | **[ng/mg]** | **[ng/mg]** | **[ng/mg]** | **[ng/mg]** | **[ng/mg]** | **[ng/mg]** | **[ng/mg]** | **[ng/mg]** | **[µg/mg]** | **[ng/mg]** | **[µg/mg]** |
| 12 month | APO E4 | Fortasyn | 311,9±8,2 | ***76,5±3,5*** | ***96,2±3,4*** | ***4,6±0,2*** | ***30,9±1,5*** | ***24,2±2,7*** | 0,4±0,1 | ***175,9±5,5*** | ***0,1±0,0*** | ***2,2±0,1*** | 80,8±1,7 |
| 12 month | APO E4 | Control | 321,6±16,6 | 105,8±11,9 | 136,9±5,4 | 3,2±0,5 | 33,4±1,9 | 38,2±4,3 | 0,6±0,2 | ***183,6±11,1*** | ***0,1±0,0*** | ***2,4±0,2**** | 88,8±5,8 |
| 12 month | WT | Fortasyn | 326,6±8,7 | ***91,5±5,5*** | ***94,2±3,9*** | ***4,3±0,1*** | ***29,9±1,2*** | ***29,2±1,5*** | 0,4±0,0 | 167,7±3,9 | 0,1±0,0 | 2,0±0,0 | 83,7±1,6 |
| 12 month | WT | Control | 294,8±11,6 | 108,6±3,9 | 132,8±10,3 | 2,9±0,1 | 29,9±1,9 | 35,5±1,6 | 0,5±0,0 | 163,8±4,9 | 0,1±0,0 | 2,1±0,1 | 84,3±1,5 |
|  |  |  |  |  |  |  |  |  |  |  |  |  |  |
| 18 month | APO E4 | Fortasyn | 368,0±13,4 | ***60,6±3,6*** | ***89,3±6,2*** | ***4,7±0,2*** | ***28,4±0,7*** | ***15,2±1,4*** | ***0,2±0,0*** | 160,4±6,2 | ***0,1±0,0*** | 2,2±0,2 | 85,2±3,1 |
| 18 month | APO E4 | Control | 364,9±3,4 | ***67,8±1,3*** | ***149,5±5,4*** | 2,2±0,0 | ***28,2±0,9*** | ***17,5±0,6*** | ***0,2±0,0*** | 157,7±3,5 | 0,1±0,0 | 2,1±0,1 | 83,1±0,8 |
| 18 month | WT | Fortasyn | 365,9±7,7 | ***65,4±2,9*** | ***107,5±2,1*** | ***4,5±0,2*** | 30,1±1,0 | ***18,9±1,0*** | 0,2±0,0 | 157,8±7,3 | ***0,1±0,0*** | 1,9±0,1 | 81,6±2,4 |
| 18 month | WT | Control | 361,6±14,1 | 77,0±2,3 | 187,4±14,5 | 2,3±0,1 | 32,4±2,3 | 22,5±1,4 | 0,2±0,0 | 156,1±6,1 | 0,1±0,0 | 2,0±0,2 | 84,5±2,4 |
